# Assessing the Perception of Undergraduate Medical Students of the Educational Environment in a Central Institute of Eastern India: A Mixed-Method Study

**DOI:** 10.7759/cureus.93908

**Published:** 2025-10-05

**Authors:** Sarthak Das, Vinayagamoorthy Venugopal, Archana Malik, Pradosh Kumar Sarangi, Prerona Das, Soumi Kundu, Md. Ehtesham Ansari, Saroj Kumar Tripathy, Tanishq Kumar, Harminder Singh, Pratima Gupta, Saurabh Varshney

**Affiliations:** 1 Pediatrics, All India Institute of Medical Sciences, Deoghar, Rampur, IND; 2 Community and Family Medicine, All India Institute of Medical Sciences, Deoghar, Rampur, IND; 3 Pulmonary Medicine, All India Institute of Medical Sciences, Deoghar, Rampur, IND; 4 Radiodiagnosis, All India Institute of Medical Sciences, Deoghar, Rampur, IND; 5 Pharmacology, All India Institute of Medical Sciences, Deoghar, Rampur, IND; 6 Microbiology, All India Institute of Medical Sciences, Deoghar, Rampur, IND; 7 Otolaryngology, All India Institute of Medical Sciences, Deoghar, Rampur, IND

**Keywords:** eastern india, learning environment, medical education, mixed-methods, undergraduate students

## Abstract

Introduction

Understanding students’ insight into the learning environment is crucial for improving the quality of medical education. Most studies evaluating medical educational environments have been conducted in well-established colleges or in developed countries with mature infrastructure and academic traditions. Evidence from newly established medical institutes in India remains scarce. This study was undertaken in a central institute of national importance in Eastern India, established in 2019 by the Government of India. Capturing student perceptions in such a formative phase provides crucial insights into strengths and areas needing improvement. The findings offer novel benchmarks for similar emerging institutes across the country.

Methods

An explanatory sequential mixed-methods model was applied. In the quantitative phase, a cross-sectional survey using the 28-item Johns Hopkins Learning Environment Scale (JHLES) was administered to all undergraduate medical students and interns via email, WhatsApp, and classroom visits. In the qualitative phase, four focus group discussions (FGDs) were conducted using a pilot-tested interview guide. Discussions were held in Hindi, audio-recorded with consent, and analyzed thematically after reaching data saturation. Quantitative data were evaluated using descriptive statistics, chi-square tests, and ANOVA to assess associations between JHLES scores and sociodemographic variables (SPSS v26; p < 0.05 considered significant). Qualitative data from transcribed FGDs were evaluated thematically using a manual, inductive approach aligned with JHLES domains. Integration of results was achieved through a joint display, enabling side-by-side correlation of quantitative scores with qualitative perceptions for an in-depth understanding of the learning environment.

Results

Out of 460 students, 434 participated in the study, yielding a response rate of 94%. The mean overall JHLES score was 87/140. More recent batches reported significantly higher scores, particularly in domains such as meaningful engagement, faculty relationships, and academic climate. Male students scored higher than females (p = 0.03). Qualitative findings supported the quantitative results and highlighted improvements in faculty interaction, mentorship, peer collaboration, and access to extracurricular activities. Variations across batches were attributed to the evolving institutional infrastructure and the development of support systems.

Conclusion

The study highlights steady advancements in the educational environment, especially among more recent student cohorts. An encouraging, welcoming, and well-organized learning environment positively affects student perception and engagement. Institutional commitment to regular assessment and targeted interventions is key to sustaining a high-quality learning environment.

## Introduction

The learning environment (LE) includes the physical, cultural, and psychosocial context in which learning takes place. It plays a key role in shaping medical undergraduate students’ attainment of the cognitive, psychomotor, and affective domains of professional identity development. Students’ interactions with their peers, faculty, institutional stewardship, and the design and implementation of the curriculum collectively reflect their perception of the learning ecosystem [1,2].

A constructive and supportive LE has been consistently associated with better academic outcomes, increased motivation, and improved student well-being. Conversely, students who perceive their LE as hostile or unsupportive may experience reduced academic efficiency and heightened levels of stress and frustration [3]. Thus, understanding and strengthening the LE is crucial for enhancing the quality of medical education and ensuring the success of future medical professionals.

Most of the existing literature evaluating students’ perceptions of the medical educational environment has been conducted in well-established medical colleges in India or in institutions from developed countries. These settings usually have long-standing academic traditions, stable faculty strength, and mature infrastructure. In contrast, there is a scarcity of evidence from newly established medical institutes in India that are still in the formative phase of their academic and infrastructural development [1-3].

This study was conducted at a central institute in Eastern India, established in 2019 by the Ministry of Health and Family Welfare (MOHFW), Government of India (GOI), and recognized as an Institute of National Importance. As a recently established institution, it is in the formative stage of academic and infrastructural growth.

To the best of our knowledge, no previously published study has captured undergraduate students’ perceptions of the educational environment in a newly established central institute of national importance in India. Hence, this study provides novel insights into the educational climate of a medical institution at an early stage of its growth, which can serve as a benchmark for improvement and a reference for other emerging institutes across the country.

This study aimed to assess the educational environment using the Johns Hopkins Learning Environment Scale (JHLES), identify associated influencing factors, and explore students' perceptions of both positive and challenging aspects of their academic experience, including their suggestions for improvement. The 28-item JHLES was used as it is a validated tool specifically designed to comprehensively measure medical students’ perceptions of the LE across multiple critical domains. The outcomes are anticipated to provide a relevant appraisal for institutional stakeholders to guide continuous improvement in the quality of medical education.

## Materials and methods

Study design and setting

A mixed-methods approach using an explanatory sequential design was adopted. A cross-sectional analytical survey (quantitative component) was followed by a descriptive qualitative research design using focus group discussions (FGDs, qualitative phase). The study was conducted at an institute of national importance located in Jharkhand.

Study participants and sampling strategy

All enrolled undergraduate medical students from all professional years, along with medical interns, were included in the study. Students who filled out the questionnaire incompletely or submitted forms with missing essential data were excluded. A complete enumeration approach was used to enroll participants in the study.

Brief procedure

In the initial step of the survey, all eligible undergraduate medical students and interns were invited to participate through various platforms such as institutional email, WhatsApp groups, and in-person classroom visits. This strategy ensured wide dissemination and increased participation within the designated timeline. The survey, forming the quantitative phase, collected demographic information and responses to the Johns Hopkins Learning Environment Scale (JHLES). The 28-item JHLES aids medical educators in identifying areas for improvement by evaluating seven domains or subscales: community of peers, faculty relationships, academic climate, meaningful engagement, mentoring, inclusion and safety, and physical space [3].

FGDs were conducted by a team of investigators experienced in qualitative research methods. A pilot-tested interview guide with probes was used to conduct the FGDs. All interviews were conducted in the vernacular Hindi language, and each session lasted approximately 40-50 minutes. The interviews were conducted in a comfortable setting for participants. Students who were willing and articulate in sharing their views were purposively selected for the FGDs from all batches. All interviews were audio-recorded after obtaining consent from participants for recording, transcription, and data extraction. Field notes were taken during the sessions. The essence of each interview was paraphrased and confirmed with respondents. Data saturation was reached after the fourth FGD; hence, no further interviews were conducted.

Statistical analysis

Quantitative Analysis

Independent variables were analyzed using descriptive statistics. Overall and domain scores of the JHLES were calculated by summing item responses. Means, standard deviations, and 95% confidence intervals were computed. Scores were also expressed as a percentage of the maximum possible, with 0-33% = poor, 34-66% = moderate/fair, and 67-100% = good/very positive perceptions.

To evaluate associations between JHLES scores and sociodemographic variables, cross-tabulations were performed. Statistical significance between independent variables and JHLES scores was assessed using t-tests and ANOVA, as applicable. The assumptions of these tests and the effect sizes were checked and reported.

In the multivariable linear regression model, independent variables were coded as follows: age was treated as a continuous variable (in years); gender was coded as a binary variable with male as the reference (male = 0, female = 1); MBBS batch was entered as an ordinal variable coded numerically by year of admission, with the most recent batch coded as 1 and progressively older batches assigned higher values; and college endorsement was included as a categorical variable with three levels, where “positive endorsement” served as the reference category, and two dummy variables were created for “neutral vs. positive” and “negative vs. positive.”

A sensitivity analysis was also conducted, excluding the college endorsement variable. All analyses were performed using IBM SPSS Statistics software (version 26) [4], with a p-value < 0.05 considered statistically significant.

Qualitative Analysis

The recorded interviews were translated from Hindi into English and transcribed verbatim. The transcripts were prepared by the investigator familiar with both languages. Two investigators carried out the translations, and their accuracy was verified. Manual thematic content analysis was then performed.

The inductive evaluation was guided by predefined (a priori) themes derived from the JHLES domains, while also allowing new themes to emerge from students’ responses. This method effectively documented the complexity of qualitative data, allowing the categorization of homogeneous responses across study participants and helping researchers link findings to the areas being explored. The guidelines of the UCLA Center for Health Policy Research were followed for analysis.

The process began with the primary investigators (first and second authors) reviewing each transcript several times. They independently coded key content, analyzed the interpretation of each sentence, and organized the derived explanations into conceptual principles regarding students’ experiences. Codes related to comparable domains were grouped to form categories, and homogeneous categories were then combined to form overarching themes for detailed exploration.

Differences in emerging codes, categories, and themes among investigators were discussed and resolved. Finally, the primary investigator reviewed the concepts and themes with the other author to clarify and reach consensus. Statements in italics indicate direct quotations or verbatim excerpts from students. These quotations support or expand upon the results and help illustrate students’ perspectives. The “Consolidated Criteria for Reporting Qualitative Research (COREQ)” guidelines were adhered to while reporting this qualitative work.

Integrative Analysis of Quantitative and Qualitative Data

The integration of qualitative and quantitative findings was achieved through the use of a joint display, allowing for parallel comparison of JHLES results and themes emerging from the FGDs. This integrative method facilitated a comprehensive understanding of the learning environment, demonstrating that the combined interpretation provided richer insights than either method alone.

The joint display served as a pictorial tool to harmonize qualitative narratives with corresponding quantitative scores, enabling deeper understanding of the reasons behind student responses. The qualitative data enhanced the quantitative findings by explaining the numerical scores and providing students’ perspectives on underlying factors. Additionally, qualitative analysis revealed how students’ sense of pride and perceptions of their role within the institution influenced their responses to JHLES items. Students also articulated their expectations and preferences regarding the learning environment during the FGDs.

Ethical considerations

This study received ethical approval from the Institutional Ethics Committee of the study setting (IEC Code: 2024-390-IND-04). Informed consent was obtained from all participants prior to their involvement. Electronic consent was secured for participants in the quantitative component, while written consent was obtained for the qualitative component.

## Results

Overall, 434 students participated in the survey out of 460, yielding a response rate of 94%; 298 (68.7%) were male. The majority of students, 199 (45.9%), belonged to the eastern region, while the fewest, 28 (6.4%), were from the central part of the country. Of the total participants, 117 (27%) were from the 2022 batch and 50 (11.5%) from the 2019 batch. The highest proportion of students was from the fifth semester (121; 27.8%), followed by the third semester (106; 24.4%), with the lowest representation from the internship (50; 11.5%). Among them, 170 (39.2%) reported that they would recommend the college to others, while 49 (11.3%) stated that they would not (Table 1).

**Table 1:** Sociodemographic profile of the study participants (N = 434). Data are presented as mean (SD) for normally distributed continuous variables, median (IQR) for skewed continuous variables, and frequency (%) for categorical variables.

Characteristics	Mean (SD) / Frequency (%)
Age (years)	21.7 (1.8)
Gender	
Male	298 (68.7)
Female	136 (31.3)
Region of India	
North	86 (19.8)
East	199 (45.9)
West	86 (19.8)
South	35 (8.1)
Central	28 (6.4)
Year of Admission	
2019	50 (11.5)
2020	63 (14.5)
2021	100 (23.0)
2022	117 (27.0)
2023	104 (24.0)
Semester	
Third	106 (24.4)
Fifth	121 (27.8)
Sixth	94 (21.6)
Ninth	63 (14.5)
Internship	50 (11.5)
College Endorsement	
Would recommend	170 (39.2)
Neutral	215 (49.5)
Would not recommend	49 (11.3)
Exam Scores	
10th standard	90.0 (9.0)
12th standard	87.9 (8.4)
MBBS Professional Exam Scores	
I (n = 351)	63 (59.6-66)
II (n = 121)	64 (60-66.1)
III (Part I) (n = 39)	62 (60-65)
III (Part II) (n = 55)	63 (59-65)

The mean overall JHLES score was 87.6, corresponding to 62.6% of the maximum possible score, indicating a moderate perception of the learning environment among participants. Among the domains, Physical Space had the highest mean score (6.9; 69% of maximum), suggesting a relatively more positive perception of facilities. Faculty Relationships also scored well (19.7; 65.7% of maximum), reflecting favourable interactions with faculty. In contrast, Inclusion & Safety had the lowest mean score (7.6; 50.7% of maximum), indicating perceived areas for improvement in inclusivity and safety. Community of Peers (19.2; 64% of maximum) and Academic Climate (15.9; 63.6% of maximum) reflected moderate perceptions (Table 2).

**Table 2:** Average scores of study participants on the JHLES and its domains (N = 434). JHLES: Johns Hopkins Learning Environment Scale.

JHLES Domain	Mean ± SD	95% CI	Maximum Possible Score	% of Maximum Possible Score
Overall	87.6 ± 16.0	86.1-89.1	140	62.6
Community of Peers	19.2 ± 4.5	18.8-19.6	30	64
Faculty Relationships	19.7 ± 5.4	19.2-20.2	30	65.7
Academic Climate	15.9 ± 3.9	15.5-16.3	25	63.6
Meaningful Engagement	12.1 ± 3.3	11.8-12.4	20	60.5
Mentoring	6.2 ± 0.9	6.1-6.4	10	62
Inclusion and Safety	7.6 ± 3.1	7.3-7.9	15	50.7
Physical Space	6.9 ± 1.6	6.7-7.1	10	69

Male students reported significantly higher overall JHLES scores than female students (88.7 vs. 85.4; p = 0.03; Cohen’s d = 0.21). Among the domains, males scored significantly higher in Community of Peers (19.8 vs. 17.5; p < 0.001; d = 0.53), whereas females reported slightly higher scores in Inclusion & Safety (8.0 vs. 7.4; p = 0.05; d = -0.20). No statistically significant gender differences were observed for Faculty Relationships, Academic Climate, Meaningful Engagement, Mentoring, or Physical Space. Overall, the effect sizes indicated a moderate difference in peer community and small differences in other domains. Tests of normality (Shapiro-Wilk) and homogeneity of variances (Levene’s test) were satisfied for all domains, except Inclusion & Safety, where Welch’s t-test was applied due to slightly unequal variances. Overall, t-test assumptions were adequately met for all comparisons (Table 3).

**Table 3:** Associations between gender and overall and domain scores of the JHLES (N=434). #p-values are based on independent-samples t-tests; p < 0.05 indicates statistical significance. JHLES: Johns Hopkins Learning Environment Scale.

Characteristics	Male Mean (SD)	Female Mean (SD)	Test statistic	p-value^#^	Cohen’s d (Effect size)
Overall score	88.7 (16.8)	85.4 (13.9)	2.15	0.03*	0.21
Community of peers	19.8 (4.3)	17.5 (4.5)	5.12	<0.001*	0.53
Faculty relationships	19.9 (5.5)	19.1 (5)	1.66	0.09	0.15
Academic climate	15.9 (3.9)	15.8 (3.8)	0.36	0.71	0.03
Meaningful engagement	12.1 (3.4)	11.9 (2.9)	0.5	0.61	0.06
Mentoring	6.3 (1.9)	6.1 (2.1)	1.28	0.20	0.10
Inclusion and safety	7.4 (3)	8 (3.1)	-1.9	0.05	-0.20
Physical space	7 (1.7)	6.9 (1.5)	0.57	0.56	0.06

Overall JHLES scores were higher among more recently enrolled students, with first-year students (2023 cohort) reporting a mean of 88.5 (SD 14.1) compared to 82.3 (SD 21.3) in the 2019 cohort, although the overall difference was marginally non-significant (F = 2.32, p = 0.06, η² = 0.021). Significant differences were observed in Faculty Relationships (F = 4.01, p = 0.003, η² = 0.036) and Meaningful Engagement (F = 3.94, p = 0.004, η² = 0.035), with recently joined students reporting higher scores. Inclusion & Safety also differed across years (F = 4.47, p = 0.001, η² = 0.040), being rated highest by junior students. No significant differences were found for Mentoring or Physical Space. Effect sizes were small to moderate, indicating a modest influence of recency of enrollment on perceptions of the learning environment. Tests of normality (Shapiro-Wilk) and homoscedasticity (Levene’s test) were satisfied for all domains (Table 4).

**Table 4:** Associations between academic year and overall domain scores of the JHLES (N=434). #p-values are based on one-way ANOVA. False Discovery Rate (FDR)-adjusted p-values were calculated using the Benjamini-Hochberg procedure. p < 0.05 indicates statistical significance. JHLES: Johns Hopkins Learning Environment Scale.

JHLES Domain	2019 Mean (SD)	2020 Mean (SD)	2021 Mean (SD)	2022 Mean (SD)	2023 Mean (SD)	F (df = 4)	p-value#	FDR-adjusted p-value	η² (Effect Size)
Overall Score	82.3 (21.3)	86.4 (14.8)	87.2 (16.9)	90.2 (14.3)	88.5 (14.1)	2.32	0.06	0.12	0.021
Community of Peers	17.9 (5.3)	18.7 (5.3)	19.0 (4.6)	19.5 (3.8)	19.8 (4.2)	1.68	0.15	0.2	0.015
Faculty Relationships	17.2 (6.6)	19.5 (5.1)	20.1 (5.2)	19.4 (5.0)	19.7 (5.1)	4.01	0.003*	0.01*	0.036
Academic Climate	14.8 (4.5)	15.8 (3.7)	15.7 (4.1)	16.5 (3.5)	16.1 (3.8)	1.85	0.12	0.19	0.017
Meaningful Engagement	10.7 (3.8)	11.5 (3.2)	12.2 (3.4)	12.7 (2.9)	12.2 (3.2)	3.94	0.004*	0.01*	0.035
Mentoring	6.2 (2.1)	6.0 (2.2)	6.2 (2.0)	6.6 (1.8)	5.9 (1.9)	1.51	0.2	0.23	0.014
Inclusion and Safety	8.9 (3.4)	7.7 (3.2)	6.9 (2.9)	7.2 (2.8)	7.9 (3.1)	4.47	0.001*	0.008*	0.04
Physical Space	6.6 (2.2)	7.1 (1.4)	7.0 (1.7)	6.9 (1.5)	7.0 (1.6)	0.75	0.55	0.55	0.007

Students’ overall JHLES scores differed significantly by college endorsement, with those who would recommend their college reporting the highest overall scores (96.2 ± 15.1), followed by neutral students (84.6 ± 12.0), and the lowest among those who would not recommend it (71.4 ± 17.0; F = 70.4, p < 0.001, η² = 0.246). Significant differences were observed across most domains, including Community of Peers (F = 48.9, η² = 0.185), Faculty Relationships (F = 56.9, η² = 0.209), Academic Climate (F = 52.5, η² = 0.196), and Meaningful Engagement (F = 54.3, η² = 0.201), all with FDR-adjusted p < 0.001. Interestingly, Inclusion & Safety scores were slightly higher among students who negatively endorsed the college (9.6 ± 3.5) compared to those recommending it (6.9 ± 3.0), possibly reflecting that students critical of the institution were more aware of, or sensitive to, policies, support systems, or formal safety measures, even if their overall college experience was less favourable. Effect sizes were moderate to large for most domains, indicating a strong association between college endorsement and perceptions of the learning environment. Tests of normality (Shapiro-Wilk) and homoscedasticity (Levene’s test) were satisfied for all domains (Table 5).

**Table 5:** Associations between status of college endorsement and overall and domain scores of the JHLES (N = 434). #p-values are based on one-way ANOVA. False Discovery Rate (FDR)-adjusted p-values were calculated using the Benjamini-Hochberg procedure. p < 0.05 indicates statistical significance. JHLES: Johns Hopkins Learning Environment Scale.

JHLES Domain	Would Recommend Mean (SD)	Neutral Mean (SD)	Would Not Recommend Mean (SD)	F (df = 2)	p-value#	FDR-adjusted p-value	η² (Effect Size)
Overall Score	96.2 (15.1)	84.6 (12.0)	71.4 (17.0)	70.4	<0.001*	<0.001*	0.246
Community of Peers	21.3 (4.0)	18.3 (3.9)	15.5 (4.8)	48.9	<0.001*	<0.001*	0.185
Faculty Relationships	22.2 (4.8)	18.9 (4.7)	14.4 (5.3)	56.9	<0.001*	<0.001*	0.209
Academic Climate	17.7 (3.6)	15.4 (3.2)	12.2 (4.1)	52.5	<0.001*	<0.001*	0.196
Meaningful Engagement	13.6 (3.0)	11.6 (2.8)	8.9 (3.3)	54.3	<0.001*	<0.001*	0.201
Mentoring	6.9 (1.8)	6.0 (1.8)	4.9 (2.1)	23.2	<0.001*	<0.001*	0.097
Inclusion and Safety	6.9 (3.0)	7.6 (2.7)	9.6 (3.5)	16.7	<0.001*	<0.001*	0.072
Physical Space	7.5 (1.5)	6.8 (1.4)	5.9 (2.3)	24.1	<0.001*	<0.001*	0.101

In the adjusted multivariable model (Table 6), only college endorsement was a statistically significant predictor of overall JHLES scores. Students with neutral (β = -11.3, 95% CI: -14.1 to -8.4, p < 0.001) and negative (β = -24.4, 95% CI: -28.9 to -19.9, p < 0.001) endorsements reported markedly lower JHLES scores compared with those with positive endorsement. Age, gender, and MBBS batch showed associations in the expected directions but were not statistically significant. To address potential overlap between college endorsement and the JHLES construct, a sensitivity analysis was conducted, excluding endorsement from the model. In this model, MBBS batch emerged as a significant predictor: students from older batches reported lower JHLES scores (β = -3.08, 95% CI: -5.3 to -0.78, p = 0.009). Age and gender remained non-significant predictors. The explanatory power of the model was unchanged (R² = 0.26), though residual autocorrelation was slightly more pronounced (Durbin-Watson = 1.25). The MBBS batch variable was coded as an ascending continuous variable (higher values indicating older batches), thereby serving as a proxy for the recency of enrolment. Variance inflation factors for all predictors were close to 1, indicating no evidence of multicollinearity. Visual inspection of residual plots confirmed approximate linearity and homoscedasticity, and no highly influential outliers were detected. Standardized betas are reported to facilitate comparability across predictors, with MBBS batch representing the strongest independent predictor in the sensitivity model.

**Table 6:** Prediction of overall JHLES score of the participants using adjusted linear regression analysis (N = 434). Note: Values are presented as regression coefficients (β) with 95% CI obtained from multivariable linear regression analysis. p-values < 0.05 were considered statistically significant. Model summary: R² = 0.26, R = 0.51, Durbin-Watson = 1.63 VIF: Variance inflation factor; JHLES: Johns Hopkins Learning Environment Scale.

Characteristics	Unstandardized β	95% CI for Unstandardized β	Coefficient Standard Error	Standardized β	p-value	VIF
Age (years)	0.61	-0.30 to 1.50	0.46	0.07	0.18	1.69
Female	-2.6	-5.40 to 0.30	1.45	-0.07	0.08	1.02
MBBS batch	-1.8	-3.90 to 0.20	1.03	-0.09	0.07	1.71
College endorsement						
Neutral vs. positive	-11.3	-14.10 to -8.40	1.43	-0.35	<0.001*	1.15
Negative vs. positive	-24.4	-28.90 to -19.90	2.28	-0.48	<0.001*	1.17
Sensitivity analysis excluding college endorsement (R = 0.16, R² = 0.26, Durbin-Watson = 1.25)						
Age (years)	0.55	-0.48 to 1.59	0.52	0.06	0.29	1.69
Female	-3.02	-6.30 to 0.23	1.65	-0.09	0.06	1
MBBS batch	-3.08	-5.30 to -0.78	1.17	-0.16	0.009*	1.68

Four FGDs were conducted with different batches of students admitted between 2019 and 2022. The 2022 batch included 12 participants, while the 2019 batch had 19 students. Gender distribution was nearly equal across all batches. In total, 42 students aged 19 to 25 years participated in the four FGDs (Table 7).

**Table 7:** Demographic profile of students who participated in focus group discussions (N = 42).

Characteristics	2019 (n = 9)	2020 (n = 10)	2021 (n = 12)	2022 (n = 11)	Total (N = 42)
Gender					
Male	5	4	6	5	20
Female	4	6	6	6	22
Age in years (range)	23-25	21-22	20-21	19-21	19-25

Qualitative results

As open-ended questions were used to collect data, themes were derived using a deductive approach, and categories within themes were developed through an inductive approach. The lines in italics represent verbatim quotes from the students who participated in the FGDs. Overall, six themes corresponded to the major domains of the JHLES questionnaire.

Theme 1: Community of peers

Under this theme, there were four categories: (i) support during exams, (ii) stressbusters, (iii) motivation to study, and (iv) academic guidance.

Category 1.1: Support During Exams

A female student from the 2021 batch mentioned, “My seniors helped me to plan the exam preparation.” Another student, a male from the 2022 batch, noted, “He helped me on the day before the exam to study important topics.” A female student from the 2023 batch said, “All the time, I ask my seniors during exams for guidance.” Apart from these verbatim quotes, codes with similar meanings were mentioned by 10 participants from various batches.

Category 1.2: Stressbusters 

A male student from the 2021 batch mentioned, “We maintain cordial relationships with peers and juniors.” A female student from the 2020 batch shared, “They organize our birthday celebrations in the hostel.” Another student, a female from the 2019 batch, expressed, “We also celebrate together Durga Puja, Saraswati Puja, Onam, Pongal, and Diwali festivals at the hostel.” Additionally, a male student from the 2021 batch stated, “We go on outings with our juniors.” Apart from these verbatim quotes, codes representing this category were noted by 19 participants from various batches.

Category 1.3: Motivation to Study 

A male student from the 2022 batch shared, “Whenever I feel stressed about studies, I talk with my senior, which motivates me to work.” A female student from the 2020 batch mentioned, “We discuss plans after the internship.” Another student from the 2023 batch said, “He gives tips for the entrance exam.” A male participant from the 2019 batch stated, “We discuss our issues and try to solve them together.” Additionally, a female student from the 2023 batch expressed, “We are closer to our seniors and friends; we find them more comfortable for non-academic matters and emotional needs.” Apart from these verbatim quotes, codes representing this category were noted by 17 participants from various batches.

Category 1.4: Academic Guidance

A female student from the 2020 batch mentioned, “Friends and seniors help, especially during classes and clinical postings, to clear doubts.” A male student from the 2019 batch shared, “We often help juniors when they have doubts.” Another student from the 2021 batch said, “We share online reading materials with friends.” Apart from these verbatim quotes, codes representing this category were noted by 18 participants from various batches.

Theme 2: Faculty relationships

Under this theme, there were six categories: (i) empathetic, (ii) approachable, (iii) advancing knowledge and skills, (iv) non-judgmental, (v) compassionate, and (vi) role model.

Category 2.1: Empathetic

A male student from the 2019 batch shared, “Sixty to seventy percent of the faculty are helpful and understanding.” A female student from the 2021 batch mentioned, “I prefer discussing my issues first with faculty rather than friends.” Apart from these verbatim quotes, codes representing similar meanings in this category were noted by a total of seven participants from various batches.

Category 2.2: Approachable

A student from the 2022 batch shared, “It was quite easy to contact faculty members regarding our performance in studies.” Another student from the 2021 batch mentioned, “Faculty members are available to clear our doubts.” A male student from the 2020 batch stated, “Except for a few senior faculty members, most of the assistant professors are available anytime for discussions.” Apart from these verbatim quotes, codes representing similar meanings in this category were noted by 17 participants from various batches.

Category 2.3: Advancing Knowledge and Skills

A student from the 2022 batch mentioned, “They help us in expanding our clinical knowledge.” Another student from the 2021 batch shared, “He accompanied me to a conference and helped me to present a paper.” Apart from these verbatim quotes, codes representing similar meanings were noted by seven participants from various batches.

Category 2.4: Non-judgmental

A female student from the 2022 batch shared, “They have never discriminated against anyone badly.” Another student from the 2020 batch mentioned, “Never encountered any partial behaviour of faculty towards students.” Apart from these verbatim quotes, codes representing similar meanings were noted by 10 participants from various batches.

Category 2.5: Compassionate

A student from the 2021 batch mentioned, “We also approach them about non-academic issues, and they provide support and try to help us.” Three more students gave similar statements. Another student shared, “One of our teachers helped me when my father was ill.” A female student from the 2022 batch said, “Once our madam arranged a vehicle for me to go to my hometown when I was stuck with no transport.”

Category 2.6: Role Model

A student from the 2021 batch mentioned, “I prefer him as my role model in my professional life.” Another student from the 2022 batch shared, “Sir inspired me to work hard and sincerely.” Apart from these verbatim quotes, codes representing similar meanings were noted by seven participants from various batches.

Theme 3: Academic climate

Under this theme, there were three categories: (i) curriculum, (ii) learning resources, and (iii) research.

Category 3.1: Curriculum

A student from the 2021 batch shared, “We are happy with the syllabus we learn.” Another student from the 2022 batch mentioned, “We have ample patients available in all departments to learn clinical skills.” A male student from the 2019 batch said, “Some departments allow evening postings, which are beneficial and allow a greater chance to interact with patients and learn better.” Additionally, a student from the 2020 batch stated, “Exam frequency was fine.” Apart from these verbatim quotes, codes representing similar meanings were noted by 30 participants from various batches.

Category 3.2: Learning Resources

A student from the 2022 batch shared, “We had access to academic fests, conferences, quizzing clubs, workshops, etc.” Another student from the 2021 batch mentioned, “We have separate museums in each department to facilitate learning by directly seeing the specimens.” Additionally, a student from the 2020 batch noted, “There is a limited number of reference books available, and also limited online literature material.” Apart from these verbatim quotes, codes representing similar meanings were noted by 12 participants from various batches.

Category 3.3: Research

A student from the 2021 batch mentioned, “We have a provision for research allowance.” Another student from the 2022 batch shared, “Some faculty members also inspired us to take up several research activities.” Apart from these verbatim quotes, codes representing similar meanings were noted by five participants from various batches.

Theme 4: Meaningful engagement

This theme had one category under the curriculum.

A student from the 2022 batch mentioned, “Some departments allow evening postings, which are beneficial and allow a greater chance to interact with patients and learn better.” Another student from the 2023 batch stated, “We are involved in establishing forums like cultural clubs and quizzing clubs.” Apart from these verbatim quotes, codes representing similar meanings were noted by seven participants from various batches.

Theme 5: Mentoring

This theme had two categories.

Category 5.1: Mentor-Mentee Program

A student from the 2021 batch shared, “I had more interaction with my first-year mentor, especially towards the end of the first year, during my professionals.” Another student from the 2022 batch mentioned, “My mentor has kept in touch with me, and I regularly update my mentor about my academic or other problems.” Apart from these verbatim quotes, codes representing similar meanings were noted by 12 participants from various batches.

Category 5.2: Challenges

A student from the 2022 batch mentioned, “Sir, the allotment is made randomly, and sometimes we are allotted mentors from senior years whom we have never met and do not know. So, it becomes difficult to communicate and approach.” Another student from the 2019 batch shared, “The mentor-mentee program was not functional for our batch.” Apart from these verbatim quotes, codes representing similar meanings were noted by five participants from various batches.

Theme 6: Inclusion and safety

This theme had one category.

Category 6.1: Safety and Non-Discrimination

A student from the 2021 batch mentioned, “There have been no issues related to safety so far.” Another student from the 2022 batch shared, “No discrimination or harassment was faced.” A participant from the 2020 batch stated, “No ragging history as well.” Additionally, a student from the 2019 batch noted, “Single rooms are provided, so privacy is also adequate.” Apart from these verbatim quotes, codes representing similar meanings were indicated by 36 participants from various batches.

Theme 7: Physical space

Under this theme, there were two categories.

Category 7.1: Personal

A male student from the 2023 batch mentioned, “Single rooms are provided, so privacy is also adequate.” Another student from the 2019 batch shared, “The canteen and cafeteria are not functional.”

Category 7.2: Infrastructure

Five students from multiple batches mentioned, “The library should be open around the clock,” and “The library was functional between 9 AM and 5 PM, so I wondered how to use it during working hours.” These statements reflected issues related to the physical infrastructure of the campus.

Most of the qualitative findings were aligned with the quantitative results. The hindering factors mentioned by the students were mainly contributed by the 2019 batch, while a few students from other batches also commented on specific concerns, particularly related to the library and mess food.

The qualitative insights were consistent with the quantitative data and are jointly displayed in Table 8.

**Table 8:** Integrated presentation of quantitative findings and deductive qualitative insights from focus group analysis. Note: Numbers in parentheses indicate the number of students who contributed to similar codes.

JHLES domain	Quantitative signal (Mean ± SD)	Representative quote(s) [Batch/Gender]	Meta-inference / explanation
Community of peers	Male: 19.8 ± 4.3; Female: 17.5 ± 4.5; 2019: 17.9 ± 5.3; 2023: 19.8 ± 4.2	“We go on outings with our juniors.” (2023, Male) “I ask seniors during exams for guidance.” (2022, Female)	Junior students report stronger peer support due to organized mentoring, cultural activities, and better infrastructure, explaining higher scores in recent batches and among males.
Faculty relationships	Male: 19.9 ± 5.5; Female: 19.1 ± 5.0; 2019: 17.2 ± 6.6; 2023: 19.7 ± 5.1	“Faculty members are available to clear doubts.” (2023, Female) “One Sir helped me when my father was ill.” (2022, Male)	Approachability, mentorship, and personalized support explain higher scores among recent cohorts and positive endorsers; gaps in early batches account for lower 2019 scores.
Academic climate	2019: 14.8 ± 4.5; 2023: 16.1 ± 3.8	“We have ample patients available to learn clinical skills.” (2023, Male) “Exam frequency was fine.” (2022, Female)	Enhanced clinical exposure, structured curriculum, and co-curricular support in newer batches drive higher engagement and positive climate perception.
Meaningful engagement	2019: 10.7 ± 3.8; 2023: 12.2 ± 3.2	“We are involved in establishing forums like cultural clubs and quizzing clubs.” (2023, Female)	Junior cohorts benefit from extracurricular and clinical opportunities that increase engagement, explaining higher scores among recent students and positive endorsements.
Mentoring	2019: 6.2 ± 2.1; 2023: 6.6 ± 1.8	“I regularly update my mentor about academic problems.” (2023, Female) “The mentor-mentee program was not functional for our batch.” (2019, Female)	Active mentoring programs in recent batches improve perception scores; non-functional programs in 2019 explain lower scores.
Inclusion and safety	Female: 8.0 ± 3.1; 2019: 6.9 ± 2.9; 2023: 7.9 ± 3.1	“There have been no issues related to safety so far.” (2023, Male) “We are restricted in many activities in the name of safety.” (2019, Male)	Positive safety policies are recognized even by critical students; higher female scores reflect greater sensitivity to institutional safety measures.
Physical space	2019: 6.6 ± 2.2; 2023: 7.0 ± 1.6	“Single rooms are provided, so privacy is adequate.” (2023, Male) “The canteen and cafeteria are not functional.” (2019, Female)	Improved infrastructure in recent batches explains higher scores; older cohorts experienced limitations in physical resources.

## Discussion

Evaluating the learning environment (LE) is crucial for enhancing medical students' professional development, knowledge acquisition, and skill-building. This mixed-methods study investigated medical students' perceptions of the LE at the All India Institute of Medical Sciences Deoghar (AIIMS Deoghar), a leading central institute established by the Ministry of Health and Family Welfare in the eastern part of India in 2019. This is the pioneering study in the Indian context that employs an integrated mixed-methods approach to assess the educational environment among medical undergraduates.

In our study, the overall average JHLES score was 87 out of a maximum of 140. Notably, the tool lacks a definite threshold to categorize scores as positive or negative. As compared to the initial validation study, which described an average score of 107, our findings are considerably lower. This disparity may stem from the initial study's single-institution design, limiting its generalizability, and from differences in student backgrounds. In the U.S., medical undergraduates typically hold a prior bachelor’s degree, while in India, students enter medical school immediately after high school. Furthermore, the hidden curriculum, shaped by institutional culture, was not considered in the initial study. However, our results are similar to other studies from Malaysia, India, and Pakistan, where JHLES scores ranged between 81.1 and 87 [5-8].

Male students reported higher mean JHLES scores than female students (88.7 ± 16.8 vs. 85.4 ± 13.9; p = 0.03), with notable differences in mentorship, peer support, and academic climate. Female students perceived unmet expectations and fewer opportunities for faculty engagement. FGDs emphasized shared concerns regarding gender-related differences in learning experiences and institutional interactions [9,10].

The first batch of students (2019) reported lower JHLES scores (82.3 ± 21.3), whereas the 2022 and 2023 batches had higher scores (90.2 ± 14.3 and 88.5 ± 14.1), reflecting the institution’s positive progression. Improvements in faculty engagement, mentorship, academic resources, and student support networks probably contributed to this upward trend. Focus group results echoed these developments. In contrast, other studies have reported declining perceptions among senior students, likely due to static support networks, emphasizing the importance of continuous institutional growth in shaping student satisfaction and learning experiences [8-11].

In this study, more recent batches reported higher scores in the domain of community of peers, indicating an improvement in peer association and support. Initiatives such as cultural clubs and academic forums have facilitated meaningful interpersonal collaboration among students. This upward trend aligns with findings from the FGDs, where newer students shared positive experiences regarding camaraderie, cooperation, and peer engagement. These results contrast with studies that have documented a decline in peer-related engagement among senior students, possibly due to heavier academic responsibilities or less structured peer interaction in established institutions [12,13]. However, a few other studies have also reported an upward trend in peer engagement, especially in institutions that have recently implemented student-driven initiatives and co-curricular platforms [11].

The study revealed notable disparities in students' perceptions of faculty relationships across batches. Male students reported higher scores in this domain compared to female students, a statistically significant difference (p = 0.03). Qualitative data supported this finding, with students from recent batches expressing greater satisfaction with faculty accessibility and responsiveness. The progressive improvement in faculty-student interaction at our newer institution may explain this trend. Conversely, other studies have shown that established colleges often struggle to maintain consistent faculty engagement over time [8,11,14,15].

In our study, male and female students reported similar scores in the academic climate domain. However, students from the 2019 batch demonstrated lower scores than those from more recent batches, suggesting improvements in the educational environment over time. Students appreciated the structured syllabus, clinical exposure, and access to educational events and departmental museums. Nonetheless, concerns were raised regarding limited resource materials, insufficient digital opportunities, and delays in institutional subscriptions. While this positive trend aligns with studies reporting enhanced academic environments in newer institutions, other research has observed a decline over time due to academic stagnation or less responsive support networks [8,10,14-17].

Students from the inaugural 2019 cohort reported noticeably lower scores in the meaningful engagement domain compared to those admitted in recent years (2022 and 2023). This reflects a favorable upward trend, likely driven by the development of student support systems, expanded extracurricular opportunities, and better-structured platforms for student participation. These findings contrast with other studies in which senior students typically report higher engagement levels. Such divergence may stem from our institution’s early growth phase, during which active improvements are ongoing. In contrast, more established institutions may struggle to maintain or adapt long-standing support systems to evolving student needs [8,11,13,17].

Mentorship is a crucial component of the LE, as emphasized in earlier studies [16-18]. The importance of student-faculty collaboration and the growing involvement of faculty in supporting students’ academic journeys were also echoed in the qualitative data from this study. Students provided diverse insights into faculty support, aligning with previous research highlighting variability in mentoring experiences [18,19]. Our findings further reinforce this evidence, showing that while some students felt well supported, others identified gaps in mentorship and engagement. Moreover, a previous study reported that many faculty members may not be adequately prepared to provide the type of mentorship most effective for student development [20], echoing the concerns voiced by participants in our FGDs.

The inclusion and safety domain demonstrated significant progress among students from the more recent admission batches. Students from the 2019 batch, who reported lower scores compared to those from 2022 and 2023, later expressed a greater sense of familiarity and perceived fairness. FGDs revealed that students recognized several new initiatives aimed at promoting gender equality, effective communication, and supportive reporting mechanisms. This upward pattern corresponds with the institution's evolving policy initiatives and student-centered transformation, which focus on fostering a more inclusive and psychologically safe academic environment [17,19,21].

Although not comprehensively discussed in the narrative interviews, students who joined within the last three years expressed greater satisfaction with the physical infrastructure, including classrooms, library spaces, and hostel facilities. This highlights the institution’s development over the years and its commitment to upgrading the physical LE. Students reported that improved facilities contributed positively to their academic experience, further reinforcing the upward trend in JHLES scores among successive student batches [8,18,20-22].

Strengths and limitations

A significant strength of this study lies in its robust mixed-methods design, which enabled an integrative understanding of students’ perceptions of their LE. By combining quantitative data with qualitative insights, the study not only captured measurable trends but also explored the underlying experiences and emotions of students. Another notable strength is the inclusion of a large, diverse sample across multiple academic years, offering a broad range of perspectives from both junior and senior cohorts. While themes were primarily mapped deductively to the JHLES domains, inductive coding was also undertaken to identify novel insights not captured by the original framework, thereby reducing potential confirmation bias.

This study has certain limitations. The use of convenience sampling and a single-institution setting may restrict the representativeness and generalisability of the findings to other academic, cultural, or geographical contexts. The strong association observed between college endorsement and overall JHLES scores may reflect common-method bias or overlapping constructs, suggesting that endorsement should be interpreted as a correlate rather than a causal predictor. Future research should consider alternative measures of institutional perception. In the qualitative component, relatively few negative perceptions were expressed, which may either represent students’ genuine experiences or result from the voluntary participation of more vocal individuals, introducing potential selection bias. Nevertheless, the large sample size, methodological rigour, and integration of quantitative and qualitative data strengthen the transferability of the findings and provide meaningful insights for comparable educational contexts.

## Conclusions

This mixed-methods study provides a comprehensive assessment of undergraduate medical students’ perceptions of the educational environment at a central institute in eastern India. The findings reveal an overall positive trend, particularly among more recent batches, reflecting institutional growth and enhanced student support systems. Domains such as meaningful engagement, faculty relationships, and community of peers showed notable improvements, as supported by both quantitative scores and qualitative insights. However, certain challenges remain, including the need for expanded digital resources and more consistent mentorship. The integration of quantitative and qualitative findings underscores the importance of cultivating a responsive, student-centered academic environment. This study offers valuable implications for institutional development while situating its contribution within the broader literature from low- and middle-income settings. Future research should focus on tracking longitudinal changes and evaluating targeted interventions. Overall, these results provide meaningful direction for ongoing institutional development and may inform similar initiatives across other emerging medical colleges in India.
